# The role of vitamin D in menopausal women’s health

**DOI:** 10.3389/fphys.2023.1211896

**Published:** 2023-06-12

**Authors:** Zhaojun Mei, Hong Hu, Yi Zou, Dandan Li

**Affiliations:** ^1^ Luzhou Maternal and Child Health Hospital (Luzhou Second People’s Hospital), Luzhou, Sichuan, China; ^2^ Department of Gynaecology, Luzhou Maternal and Child Health Hospital (Luzhou Second People’s Hospital), Luzhou, Sichuan, China; ^3^ Department of Nephrology, Luzhou Maternal and Child Health Hospital (Luzhou Second People’s Hospital), Luzhou, Sichuan, China; ^4^ School of Basic Medical Sciences, University of Chinese Academy of Sciences, Beijing, China

**Keywords:** vitamin D, menopause, osteoporosis, cardiovascular disease, genitourinary syndrome of menopause

## Abstract

Vitamin D (VD) is known to play an important role in the maintenance of calcium homeostasis and bone metabolism. In recent years, there has also been a growing interest in Vitamin D for health issues beyond the bones. Menopausal women are at risk of reduced bone density and increased risk of fracture due to a decline in estrogen levels. There is also an increased risk of cardiovascular disease, diabetes and hyperlipidaemia due to impaired lipid metabolism. The menopausal and emotional symptoms due to menopause are also increasingly prominent. This article summarizes the role of Vitamin D in menopausal women’s health, including the effects of Vitamin D on skeletal muscle, cardiovascular disease, Genitourinary Syndrome of Menopause (GSM), cancer and emotional symptoms. Vitamin D regulates the growth of vaginal epithelial cells and alleviates genitourinary tract problems in menopausal women. Vitamin D also modulates immune function and influences the production of adipokines. Vitamin D and its metabolites also have an anti-proliferative effect on tumour cells. This narrative review, by summarizing recent work on the role of Vitamin D in menopausal women and in animal models of menopause, aims to provide a basis for further development of the role of Vitamin D in the health of menopausal women.

## 1 Introduction

As the population ages, the problems associated with menopause in women become more pronounced (Mosconi et al., 2021). As a result of ovarian failure, estrogen declines in menopausal women, leading to short-term menstrual disorders, hot flushes and night sweats, sleep disturbances, and loss of libido, and long-term increased risk of osteoporosis and cardiovascular disease (Matyjaszek-Matuszek et al., 2015). Estrogen therapy is an important option for menopausal women. There is still much debate about the long-term use of estrogen in menopausal women, especially in those at high risk of developing breast cancer, endometrial cancer and cardiovascular disease (Sturdee et al., 2011). Therefore, non-hormone replacement therapy is of increasing concern.

VD is a fat-soluble vitamin that includes VD_2_ and VD_3_. VD_2_ is of plant origin. VD_3_ is synthesised from 7-dehydrocholesterol in human skin by sunlight exposure and is converted to the biologically active 1,25(OH)_2_D_3_ by the action of liver, kidney and mitochondrial hydroxylases (Vacek et al., 2012). VD mainly regulates calcium and phosphorus metabolism and promotes bone growth. There is growing interest in the important role of VD outside the skeleton, including regulation of immune function, regulation of cell growth, and reduction of oxidative stress and tissue damage (Matta Reddy et al., 2022). Studies have linked VD deficiency to cardiovascular disease, metabolic syndrome, cancer and immune system disorders (Sutton and MacDonald, 2003; Rosen, 2011). The Institute of Medicine (IOM) considered a 25(OH)D level below 20 ng/mL to be considered VD deficiency. However, there is no agreement between institutions on the ideal 25(OH)D level. The Endocrine Society defined 25(OH)D concentrations of 30 ng/mL as the lower limit of normal, and 21–29 ng/mL as inadequate and <20 ng/mL as deficient. About one billion people worldwide are VD deficient (Holick, 2007). It is worth noting that VD deficiency is widely prevalent among menopausal women, accounting for about 50%–80% of the total number of menopausal women. (Kennel et al., 2010; Samuel and Borrell, 2013). In menopausal women, the ability of the skin and kidneys to produce 1,25(OH)_2_D_3_ is reduced and intestinal absorption is reduced, further contributing to lower VD levels in the body (Holick et al., 1989). In this review, we review the literature published in recent years on the role of VD in osteoporosis, cardiovascular disease and oncology in menopausal women, including randomized controlled trials, observational studies and animal studies, to summarise the potential role of VD in improving menopausal symptoms and related health outcomes.

## 2 Osteoporosis and muscle loss

The incidence of osteoporosis gradually increases with age. In menopausal women, due to a decrease in estrogen production, calcium absorption in the small intestine is reduced, ultimately leading to a decrease in bone density (de Lemos, 2004). The most important functions of VD are to promote the absorption of calcium and phosphorus from the small intestine, to promote new bone production and calcification, and to regulate parathyroid hormone to maintain blood calcium and phosphorus concentrations (Fleet, 2022). It plays a role in the prevention or treatment of osteoporosis. When VD levels are reduced, this leads to secondary hyperparathyroidism, which induces a series of changes in bone metabolism leading to reduced bone mass and osteoporotic fractures (Mosali et al., 2014). VD receptors (VDR) have been shown to be present in muscle tissue, and VD is involved in regulating the proliferation and differentiation of myoblasts, significantly improving muscle strength and function, and improving balance (Dzik and Kaczor, 2019). Myalgia, decreased muscle strength, reduced physical performance, and altered muscle morphology are common in patients with VD deficiency. A study assessed the relationship between muscle function and muscle strength in 54 postmenopausal women and found that 25(OH)D levels ≥20 ng/mL were associated with better lower limb muscle function and strength (Mastaglia et al., 2011). Calcium plus VD has been shown to reduce bone loss in perimenopausal and postmenopausal women. In an 18-year study of 72,337 postmenopausal women (Feskanich et al., 2003), adequate VD intake was found to be associated with a reduced risk of osteoporotic hip fracture in postmenopausal women. A meta-analysis demonstrated that combined calcium and VD supplementation may prevent osteoporotic hip fractures in postmenopausal women (Liu et al., 2020). VD, a calcium-regulating hormone that affects bone metabolism and calcium homeostasis, is a commonly used drug for the prevention and treatment of osteoporosis. There is considerable controversy regarding the dose of VD for the prevention and treatment of osteoporosis, and more studies should be conducted to explore the optimal dose of VD for different populations. As shown in Table 1, we summarise the effects of VD on osteoporosis and muscle loss.

**TABLE 1:** Studies of VD in osteoporosis and muscle loss.

Vitamin D	Methods	Results	Ref
VD_3_	36,282 postmenopausal women receive 1000 mg carbonate with 400 IU of VD_3_ daily or placebo for 7 years of follow-up	Calcium plus VD improved hip BMD^a^ in postmenopausal women but did not significantly reduce hip fractures	Jackson et al. (2006)
VD_3_	160 women were into the VD group (1000 IU of VD_3_/day, n = 80) or placebo group (n = 80) for 9 months	In young postmenopausal women with VD deficiency, supplementation with 1000 IU of VD_3_ alone may reduce bone turnover (s-CTX^b^, P1NP^c^) markers	Nahas-Neto et al. (2018)
Calcitriol	70 post-menopausal women were into two groups: 40 patients received calcitriol (0.5 microg/day) and calcium; and 30 patients received calcium alone for 6 months	Calcitriol treatment increased BMD and reduced serum IL-1 and TNF-alpha concentrations	Inanir et al. (2004)
Cholecalciferol and calcitriol	485 postmenopausal women were divided three group, which were treated with calcium (600 mg/d) alone, calcium and cholecalciferol (800 IU/d) or calcium and calcitriol (0.25 μg/d)	Supplementation with calcitriol and calcium modifies the bone turnover marker (β-CTX, P1NP) levels, supplementation with cholecalciferol and calcium prevents aging-mediated deterioration in quality of life	Gao et al. (2015)
Calcitriol	141 postmenopausal women were into two groups: 75 participants received calcitriol 0.5 µg/day and 66 participants received a placebo for 12 weeks	Calcitriol reduces serum PTH^d^, creatinine, uric acid and improves left hand grip strength	Cheng et al. (2018)
Calcifediol	113 post-menopausal women received calcifediol (20 μg, 4 oral drops/day) for a 6-month period for 6 months	Calcifediol improves serum levels of 25(OH)D and muscle function and reduces the average number of falls in postmenopausal women	Iolascon et al. (2017)
1alpha hydroxyVD_3_	92 osteoporotic women were into four groups: 1alpha hydroxyVD_3_, (0.75 microg/day, n = 29), VK_2_ (n = 22), VD_3_ plus vitamin VK_2_ (n = 21), and calcium (n = 20)	Combined administration of VD_3_ and VK_2_ helps to increase BMD in the lumbar spine of postmenopausal women	Iwamoto et al. (2000)
VD_3_	160 postmenopausal women were into two groups: VD group, (1,000 IU/day, n = 80) and placebo group (n = 80) for 9 months	VD supplementation alone can reduce the incidence of falls and improve postural balance in postmenopausal women	Cangussu et al. (2016)

^a^ Bone Mineral Density.

^b^ Serum C-terminal telopeptide of type I collagen.

^c^ Procollagen type 1 N-terminal propeptide.

^d^ Parathyroid Hormon.

## 3 Cardiovascular disease and glucolipid metabolism

Estrogen has a protective effect on the heart. It regulates lipid metabolism in the liver and increases the synthesis of low-density lipoprotein (LDL) receptors, thereby reducing LDL levels. Estrogen also increases the activity of lipoprotein lipase and increases high-density lipoprotein (HDL) levels (Mendelsohn and Karas, 1999). Menopause is associated with weight gain and changes in body fat distribution in women. Menopausal women suffer from lower levels of estrogen, leading to disorders of lipid metabolism and abnormal fat distribution in the body (Usategui-Martín et al., 2019). The dysregulation of lipid metabolism further leads to the development of metabolic syndromes (MetS) including cardiovascular disease, type II diabetes, and hyperlipidaemia (Janssen et al., 2002). Pro-inflammatory cytokines such as tumour necrosis factor-alpha (TNF-α) and adipocytokines play an important role in hepatic steatosis and inflammatory responses. In recent years, there has been an increasing number of studies on the effects of VD on non-classical pathways, such as VD may prevent the development of diabetes, hyperlipidaemia and cardiovascular disease (Darraj et al., 2019). VD regulates immune function and has a variety of biological effects such as anti-inflammatory and anti-oxidative stress (Lee et al., 2015). There is growing evidence that VD deficiency may lead to immune dysfunction and promote the development of cardiovascular and metabolic diseases (Martins et al., 2007; Pacini et al., 2008). A cross-sectional study found that 25(OH)D was inversely associated with fasting glucose (Alharazy et al., 2021), insulin and C-peptide in postmenopausal women with type II diabetes. VDR is expressed in the cardiovascular system, and VD inhibits the renin-angiotensin system, increases endothelial-type nitric oxide and avoids atherosclerosis (Li et al., 2002). VD regulates insulin secretion and enhances insulin sensitivity, reduces systemic inflammation thereby improving insulin resistance (Rammos et al., 2008). Cardiomyocytes have VDR and 1,25(OH)_2_D_3_-dependent calcium-binding proteins. VD has an effect on extracellular matrix remodelling, cardiomyocyte hypertrophy and proliferation (Zittermann et al., 2003). VD can also influence the production of adipokines (Rodrigues et al., 2014). Ma et al. found that serum 25(OH) D was independently and negatively associated with carotid atherosclerosis in postmenopausal women with normal blood pressure and glucose tolerance (Ma et al., 2014). Chacko et al. demonstrated that higher serum 25(OH) D concentrations may be negatively associated with obesity, triglycerides, HDL-cholesterol ratio and metabolic syndrome (Chacko et al., 2011). A cohort study found that VD deficiency in postmenopausal women was associated with a higher prevalence of MetS (Schmitt et al., 2018). Women with VD deficiency had a higher risk of MetS and LDL than those with adequate levels. There is still considerable controversy about VD attenuating cardiovascular disease in menopausal women, and some studies have reported no association between serum VD levels and MetS (de Boer et al., 2008; Guasch et al., 2012). VD deficiency is very common in menopausal women and this undoubtedly exacerbates the risk of menopause-related cardiovascular disease and dyslipidaemia. Therefore, the addition of VD to conventional therapy may be a promising treatment modality. As shown in Table 2, we summarise the studies related to VD on cardiovascular disease and abnormal glucolipid metabolism.

**TABLE 2:** Studies on the role of VD in metabolic syndrome.

Vitamin D	Methods	Results	Ref
VD_3_	600 postmenopausal women were given 1,000 mg calcium +400 IU VD_3_ and placebo respectively	Supplemental CaD significantly increases 25(OH)D concentrations and decreases LDL-C	Schnatz et al. (2014)
Cholecalciferol	160 women were randomized to 2 groups: oral 1,000 IU cholecalciferol/d (n = 80) or placebo (n = 80) for 9 months	Supplementation with 1,000 IU of VD alone was associated with an increase in adiponectin and a decrease in resistin	Schmitt et al. (2023)
VD	104 postmenopausal women with type 2 diabetes were assigned in to 2 groups: a group consuming 4000 IU VD (n = 52) or a group consuming placebo (n = 52)	Supplementation with VD (4000 IU/d) may have a beneficial effect on serum triglyceride levels	Muñoz-Aguirre et al. (2015)
VD_3_	Women undergoing VD supplementation had a lower risk of MetS, hypertriglyceridemia, and hyperglycemia for 9 months	Women undergoing VD supplementation had a lower risk of MetS, hypertriglyceridemia, and hyperglycemia	Ferreira et al. (2020)
VD_2_	80 postmenopausal women were assigned to treatment (N = 40, receiving VD_2_ 40,000 IU/week) or control (N = 40, receiving placebo) for 10 weeks	VD_2_ supplementation with ergocalciferol 40,000 IU/week can reduce hsCRP level	Indhavivadhana et al. (2022)
VD	59 postmenopausal women with type 2 diabetes received fortified yogurt (2000 IU VD in 100 g/day) or plain yogurt (PY) for 12 weeks	Daily consumption of 2000 IU VD-fortified yogurt improved glycemic markers, anthropometric indexes, inflammation, and bone turnover markers in postmenopausal women with type 2 diabetes	Jafari et al. (2016)

## 4 Genitourinary Syndrome of Menopause

Genitourinary syndrome of menopause (GSM) is a condition in which a woman experiences vaginal dryness, pain, difficulty with intercourse, recurrent vaginitis and difficulty urinating, along with frequent and urgent urination, when her ovarian function declines and her estrogen levels decrease (Mei and Li, 2022). Reduced estrogen further leads to atrophy of the vaginal wall, thinning of the epithelium, reduced glycogen content, increased pH in the vagina and increased bacterial vaginosis infections (Navaneethan et al., 2015). Estrogen therapy is one way to improve the symptoms of vaginal atrophy in post-menopausal women. However, major concerns remain about the use of estrogen in patients with breast and endometrial cancers. Vaginal atrophy is one of the most common side effects of tamoxifen use in breast cancer patients. The use of hormones for vaginal atrophy is prohibited in these women. Therefore there is a growing interest in finding safe and effective alternatives. VD is involved in regulating cell growth and differentiation (Costantino and Guaraldi, 2008), particularly in the vaginal epithelium. VD supplementation will promote squamous maturation of the vaginal epithelium, proliferation and differentiation of vaginal mucosal cells and re-establishment of the physical barrier of the vagina. Rad *et al.* demonstrated that VD vaginal suppositories improved vaginal dryness and lowered pH in women with vaginal atrophy (Rad et al., 2015). A double-blind placebo-controlled trial found that 40,000 IU of VD given weekly to 80 postmenopausal women significantly improved vaginal maturation index (VMI), vaginal pH and vaginal dryness symptoms (Kamronrithisorn et al., 2020). Recurrent urinary tract infections are another problem faced by postmenopausal women. Tight junction proteins play an important function in maintaining the integrity of the epithelial barrier. When estrogen levels decrease, accompanied by a decrease in antimicrobial peptides and barrier proteins, the permeability of the bladder urinary epithelium increases, further leading to thinning of the urinary epithelium and increasing the risk of urinary tract infections. Mohanty *et al.* found that VD induced ocludin and claudin-14 in bladder maturation surface cells (Mohanty et al., 2020), which increased intercellular adhesion and promoted epithelial integrity. A possible mechanism for the vaginal effects of VD is due to the presence of VDR in the basal cell layer of vaginal tissue (Keshavarzi et al., 2019). Lee *et al.* first revealed that VD positively regulates intercellular junctions by increasing the VDR/p-RhoA/p-Ezrin pathway (Lee et al., 2017). VD has a protective effect against vaginal atrophy in postmenopausal women and is inexpensive and without adverse effects. Therefore, in addition to topical oestrogen use, oral or vaginal VD use in post-menopausal women is very effective in reducing GSM-induced menopausal symptoms.

## 5 Cancer

The immune microenvironment has an important impact on the inflammatory status of postmenopausal women. Estrogen acts as a booster of humoral immunity and, due to the lack of estrogen in postmenopausal women, their levels of the pro-inflammatory cytokines TNF-α, IL-1 and IL-6 are elevated against pathogens (Gameiro and Romao, 2010). Human papillomavirus (HPV) infection has a second peak in postmenopausal women (Smith et al., 2008), and the vast majority (over 95%) of cervical cancers are caused by HPV. VD and its metabolites have anti-proliferative effects on tumour cells, further inhibiting tumour spread and invasion by inhibiting tumour angiogenesis and cell growth, thereby reducing the incidence of many cancers (Vanoirbeek et al., 2011). Moreover, VD can also increase the sensitivity of radiotherapy and chemotherapy (Pilz et al., 2013). It is well known that immune cells express VDR. VD activates and induces the expression of VDR in lymphocytes, while dendritic cells and macrophages constitutively express the receptor (Martens et al., 2020). Higher VD intake is associated with a reduced risk of lung, breast and ovarian cancers (Cheng et al., 2013). 1,25(OH)_2_D_3_ binds to the VDR to promote immunomodulatory and anticancer effects. Serum 25(OH) D levels were found to be significantly lower in breast cancer patients than in healthy controls (Karthikayan et al., 2018). A meta-analysis of 14 studies showed a significant negative association between serum 25(OH)D levels and breast cancer risk (RR = 0.845, 95% CI = 0.750–0.951) (Wang et al., 2013). A dose-response analysis showed a significant 3.2% reduction in breast cancer risk for every 10 ng/mL increase in serum 25(OH)D concentrations (Abbas et al., 2008). A population-based case-control study found a significant negative association between serum 25(OH)D concentrations and postmenopausal breast cancer risk. In the Women’s Health Initiative calcium + VD (CaD) trial, CaD supplementation in postmenopausal women was found to be associated with a reduced risk of breast ductal carcinoma *in situ* (Peila et al., 2021). The study further found that VD intake in non-smoking postmenopausal women was associated with a lower risk of lung cancer. Cadeau et al. evaluated the interaction between VD supplementation and menopausal hormone therapy (MHT) use during menopausa (Cadeau et al., 2016). A prospective survey of VD supplementation in 57,403 postmenopausal women found that VD supplementation may reduce the risk of breast cancer in MHT users. Serum VD levels are also associated with the presence and histologic grade of colorectal adenomas in perimenopausal and postmenopausal women. Most current studies suggest that adequate VD supplementation in perimenopausal and postmenopausal women may be beneficial in reducing cancer risk.

## 6 Climacteric and emotional symptoms

The majority of women in the ageing population spend 1/3 of their lives in a post-menopausal state, and menopausal women are significantly more likely to suffer from depression and anxiety disorders due to loss of ovarian function and low estrogen status (Arevalo et al., 2015). However, women going through menopause can experience a range of vasodilatory symptoms in the short and medium term, including: hot flushes, palpitations, night sweats, and cold hands and feet (Castanho et al., 2014). Studies have demonstrated that the status of pro-inflammatory cytokines may be higher in the periphery and hippocampus of depressed patients, and that pro-inflammatory cytokines can induce depression-like behaviours (Jeon and Kim, 2017). There is growing interest in the potential of nutrients to improve mental health and mental status in women. People with depression are usually less physically active and will spend more time indoors. VDR is located in brain regions involved in emotional processing (Lerchbaum, 2014). VD levels are low in depressed patients and supplementation has been shown to help improve mood. VD influences the production of pro-inflammatory cytokines, which in turn influence mood by activating the stress response (Kerr et al., 2015). If the central nervous system is involved, cognitive function may be affected. Annweiler *et al* (Annweiler et al., 2010) demonstrated that VD insufficiency is associated with cognitive impairment. VD has a protective and regulatory effect on the brain dopamine system, suggesting similarity to antidepressants. Zhang et al. evaluated the effects of VD and 17β-estradiol on depressive symptoms in ovarian-deviated rats (OVX) (Zhang et al., 2020). It was found that both VD and 17β-estradiol showed antidepressant-like activity in OVX rats, and exerted neuroprotective effects by reducing OVX-induced apoptosis and neuronal damage, and reducing the expression of pro-inflammatory cytokines in the hippocampus of OVX rats. A study of 81,189 members of the Women’s Health Initiative found that 400 IU of VD from food sources reduced the risk of depressive symptoms in year 3 by 20% compared to 100 IU from food sources (OR: 0.80; 95% ci: 0.67, 0.95; *p* = 0.001) (Bertone-Johnson et al., 2011). In addition to this, there is an association between VD deficiency and pelvic organ prolapse (POP) and stress urinary incontinence in postmenopausal women. In a prospective case-control study, VD levels were significantly lower in women with POP than in women without POP (Navaneethan et al., 2015).

## 7 Conclusion

In summary, VD insufficiency is a common but neglected health problem in healthy menopausal women. VD status is associated with skeletal muscle, cardiovascular disease, diabetes, GSM, and menopausal symptoms in menopausal women. VD supplementation is a safe, inexpensive treatment that plays an important role in improving the overall state of menopausal women. Therefore, it is necessary to study the effects of VD supplementation in perimenopausal and postmenopausal women. In the future, more high-quality randomised controlled trials are needed to determine optimal 25(OH)D levels and to clarify the potential adverse effects of VD and calcium supplementation.
